# Assessment of Systemic Inflammation as a Tool for Estimating the Risk of Death by Visceral Leishmaniasis

**DOI:** 10.3390/pathogens15030259

**Published:** 2026-02-28

**Authors:** Ingridi de Souza Sene, Vladimir Costa Silva, Débora Cavalcante Brás, Dorcas Lamounier Costa, Gabriel Reis Ferreira, Carlos Henrique Nery Costa

**Affiliations:** 1 LAPAC—Patologia Cirúrgica e Molecular and Mestrado em Ciências e Saúde, Centro de Ciências das Saúde, Universidade Federal do Piauí, Teresina 64049-550, Brazil; ingssene@gmail.com (I.d.S.S.); dorcas.lc@gmail.com (D.L.C.); 2 Laboratory of Genomic Surveillance and Molecular Biology (LVGBM)—Oswaldo Cruz Foundation-Piauí, Teresina 64049-550, Brazil; vladimir.costa@fiocruz.br (V.C.S.); deborabraz@ufpi.edu.br (D.C.B.); 3 Núcleo de Tecnologia Farmacêutica, Universidade Federal do Piauí, Teresina 64049-550, Brazil; 4 Centro de Inteligência em Agravos Tropicais Emergentes e Negligenciados (CIATEN) and Departamento Materno Infantil, Centro de Ciências das Saúde, Universidade Federal do Piauí, Teresina 64049-550, Brazil; 5 Centro de Inteligência em Agravos Tropicais Emergentes e Negligenciados (CIATEN) and Tropical Diseases Department of Microbiology, Infectious Disease and Immunology, Faculty of Medicine, University of Laval, Québec, QC G1V 0A6, Canada; 6 Centro de Inteligência em Agravos Tropicais Emergentes e Negligenciados (CIATEN) and Departamento de Medicina Comunitária, Centro de Ciências das Saúde, Universidade Federal do Piauí, Teresina 64049-550, Brazil

**Keywords:** visceral leishmaniasis, C-reactive protein, IL-6, biomarkers, prognosis, mortality, severity of illness index

## Abstract

Background: Visceral leishmaniasis (VL) is a life-threatening protozoan disease prevalent in tropical and subtropical regions and a frequent coinfection among people living with HIV. Early identification of patients at high risk of death may reduce case-fatality. This study evaluated the post-test prognostic value of C-reactive protein (CRP) and interleukin-6 (IL-6) as biomarkers of mortality in VL. Methods: A retrospective hospital-based cohort of 101 VL patients was analyzed. CRP and IL-6 concentrations at admission were correlated with clinical findings, the Kala-Cal^®^ prognostic score, and in-hospital mortality. Results: Eight patients died, most presenting with hemorrhagic manifestations. At admission, 87.1% of patients had both biomarkers above the predefined cut-offs. CRP and IL-6 levels were markedly elevated in patients with hemorrhage or fatal outcomes. The AUC was 0.85 for CRP and 0.87 for IL-6, with no significant difference between markers. Optimal prognostic cut-offs were 150 mg/L for CRP and 90 pg/mL for IL-6. Conclusions: In this sample, CRP and IL-6 showed good prognostic performance in VL. In patients with low initial clinical risk, positive biomarker results substantially increased the probability of death. When combined with Kala-Cal^®^, these markers may improve risk stratification and guide referral decisions.

## 1. Introduction

Visceral leishmaniasis (VL), also called kala-azar, is a disease caused by the protozoa *Leishmania infantum* and *Leishmania donovani*, transmitted by the bite of several species of sand flies. *L. donovani* is distributed mostly in South Asia and East Africa, while *L. infantum* is distributed in the rest of tropical and sub-tropical areas, except Southeast Asia and Oceania [1]. More recently, the disease has been transmitted as an urban epidemic, particularly in the South Cone of South American countries, especially in Brazil [2]. In the cities, VL met AIDS as a new coinfection, resulting in increased morbidity and mortality of both diseases [3].

Patients usually have prolonged fever, weight loss, paleness, and enlarged liver and spleen. Reduced red cells, white cells, and platelets are common, as well as reduced albumin and increased globulins, liver enzymes, and creatinine. Emaciation and jaundice are unusual but indicate severe disease. Hemorrhages and bacterial infections are the most fearful complications, frequently resulting in death [4]. Even after the diagnosis and proper treatment, the disease kills near 10% of patients [5].

These complications are thought to result from a slow-motion, overwhelming proinflammatory cytokine response followed by a immunological down-regulation, similar to bacterial sepsis [6]. Given that the biological effects of IL-6 are consistent with key disease manifestations, IL-6 has been proposed as a central mediator in disease establishment [7,8]. C-reactive protein is an acute-phase protein, released after IL-6 action. Both have been evaluated as prognostic markers for sepsis [9,10]. Given the central role of IL-6 in pathogenesis and severity in VL and the disease pathogenetic similarities with sepsis, IL-6 and CRP are candidates to be biomarkers of severity and mortality in patients with VL. In this study, their role in the prognosis and diagnosis of severe disease are evaluated.

Numerous prognostic models have been developed to help refer patients with VL to medical attention at different levels of health care, among them the Kala-Cal^®^ (2019) (version 1.0), an online software easily accessible through cell phones at the website https://www.sbmt.org.br/kalacal/ (accessed on 25 January 2026) [4]. Here, the prognostic roles of IL-6 and CRP was evaluated after clinical examination and Kala-Cal^®^ assessment for analyzing their role in guiding the referral of VL patients through the healthcare network.

## 2. Materials and Methods

### 2.1. Study Design

In a retrospective open cohort study, the clinical findings and plasma IL-6 and CRP concentrations at hospital admission were followed up to the outcomes of discharge or death.

### 2.2. Study Population

We studied 101 patients diagnosed with VL, all hospitalized at the Instituto de Doenças Tropicais “Natan Portella” in Teresina, Brazil, a regional reference hospital. Diagnosis was confirmed either by serology or direct parasitological identification of *L. infantum* amastigotes in bone marrow or positive culture. The study included all consecutively admitted patients with confirmed VL who consented to participate. The sample size was determined a priori to provide sufficient statistical power for the analysis of multiple plasma protein concentrations in patients with visceral leishmaniasis.

### 2.3. Clinical Evaluation

Clinical parameters were evaluated by the attending physician and recorded in a questionnaire. In addition to age and sex, nutritional data, various signs and symptoms, chest X-Ray, bacterial culture results, HIV testing, and hematological parameters were recorded. Hemorrhage was defined as a binary variable indicating the presence of clinically evident bleeding at one or more anatomical sites at presentation.

#### 2.3.1. Quantification of IL-6 and CRP

Plasma was analyzed by flow cytometry using the CBA^®^ inflammatory cytokine kits. The cytokines were measured following the manufacturer’s guidelines (Becton Dickinson Biosciences Pharmingen, San Diego, CA, USA). Only IL-6 results are shown in this article. The quantitative measurement of CRP was performed using the dry chemistry method (Ortho Clinical Diagnostics, Raritan, NJ, USA), according to the manufacturer’s recommendations.

#### 2.3.2. Probability of Death

The Kala-Cal^®^ software was used to estimate the probability of death [4]. The clinical version of Kala-Cal^®^ is designed to be applied at the first medical examination, when the clinical suspicion of VL is raised. In this version, the variables that accounted for estimating the chance of death are age, the number of bleeding sites, edema, jaundice, dyspnea, vomiting, suspected bacterial infection, and HIV infection. Subjects who had a chance of death greater than 20% were considered to have severe disease for the sake of data presentation.

### 2.4. Ethical Considerations

The project was approved by the Research Ethics Committee of the Federal University of Piauí (CAE: 44037015.3.0000.5314), by the precepts of the Resolution of the National Health Council-CNS 466/12, which deals with the Guidelines and Norms for Research Involving Human Subjects. All patients included in the study, or their guardians, signed an informed consent form.

### 2.5. Statistical Analysis

Data was stored and analyzed in Stata/IC 15.1 (College Station, TX, USA). Data normalization was performed using natural logarithm when necessary. Differences between dichotomous variables were analyzed using the chi-square test and Fisher’s exact test. Spearman’s correlation analysis was used to detect the correlation between continuous variables with sparse data, and Pearson’s correlation analysis was used for variables with normal distribution. Wilcoxon and Kruskal–Wallis tests compared variables without normal distribution. Multivariate logistic regression preceded the estimation of the areas under the curve.

## 3. Results

### 3.1. Study Population

Direct microscopy was positive in 84 out of 101 participants (83.4%). The mean age was 27.5 years, with 36 children and adolescents up to 18 (36.6%) and 13 infants up to 12 months of age (12.8%). Nearly a quarter of patients were under five years of age. Thirty patients (29.7%) were over the age of 40 years. Fifty-nine were males (58.4%), and 35 had HIV-1/*Leishmania* coinfection (34.5%). At admission, 18 patients (17.9%) had a chance of death estimated as over 20.0% by the Kala-Cal^®^ software, but only eight patients died while in the hospital (7.9%) (Table 1). The deaths occurred in one eight-year-old child and seven adults. Four were males and four females. Two were infected with HIV.

### 3.2. CRP

**Figure 1:**
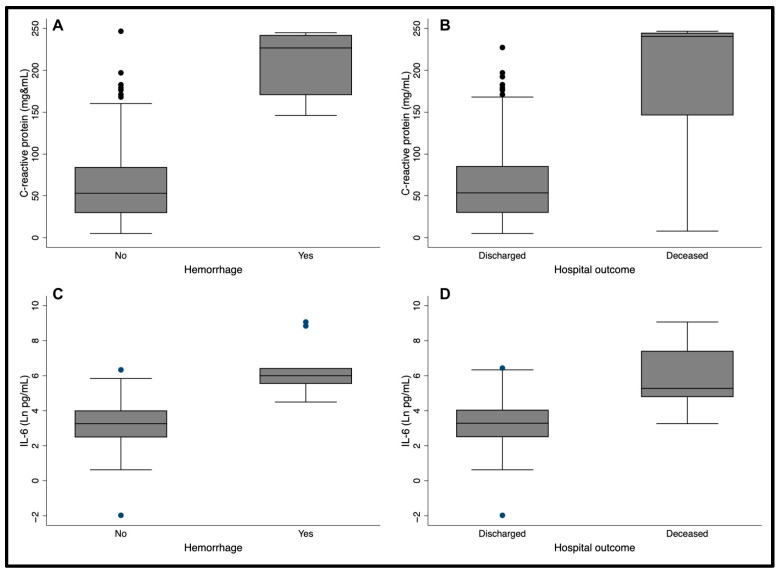
Concentration of C-reactive protein and IL-6 according to the presence of hemorrhage and the outcome of death.

**Figure 2:**
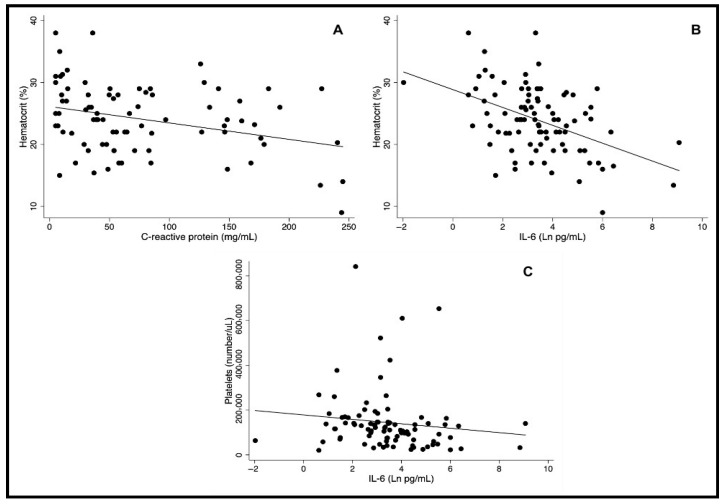
Correlation between C-reactive protein with hematocrit and of IL-6 with hematocrit and platelets.

CRP was measured in only 93 patients since the plasma volume of eight samples was unsatisfactory. Considering that the standard value of CRP is less than 10.0 mg/L in healthy individuals [11,12], 81/93 (87.1%) patients had an abnormal result. The mean and median values were 78.3 mg/L and 56.4 mg/L. The minimum detected level was 5.0 mg/L, and the maximum was 246.6 mg/L. Age and sex were not associated with CRP. However, patients with hemorrhages had a much higher CRP concentration (226.65 mg/L versus 53.30 mg/L; *p*-value < 0.001) (Figure 1A) and those who died had a median CRP approximately five times higher than those who survived (240.4 mg/L versus 53.7 mg/L; *p*-value = 0.003) (Figure 1B). Low hematocrit levels were negatively correlated with CRP (r = −0.28, *p*-value = 0.01). Conversely, individuals with anemia had higher CRP (81.9 mg/mL versus 26.9 mg/mL, *p*-value = 0.003) (Figure 2A). No other clinical manifestations were associated with the CRP concentration.

### 3.3. IL-6

IL-6 was detected in all 101 patients, considering a minimum detection plasma level was 0.13 pg/mL. Among these, 88 patients (87.1%) had plasma IL-6 levels exceeding the normal cut-off of 5.0 pg/mL [13]. The mean concentration was 227.9 pg/mL, and the median was 29.3 pg/mL. Like CRP, age and sex were also not associated with IL-6. IL-6 levels were 15 times greater in patients with hemorrhage than those without (401.23 versus 26.04; *p*-value < 0.001) (Figure 1C) and the median IL-6 concentration was over seven times higher in patients who died compared to those who survived (196.0 versus 27.0; *p*-value = 0.003) (Figure 1D). Red cell count was inversely correlated to IL-6 concentration (r = −40, *p*-value < 0.001), and patients with hematocrit lower than 30% had much higher IL-6 concentration (mean 270.6 versus 11.7 and median 30.2 versus 4.2) (Figure 2B). Platelet count was negatively and significantly correlated with IL-6 (r = −0.29, *p*-value 0.006), and individuals with thrombocytopenia (platelets < 150.000/mL) had higher IL-6 (mean 313.2 pg/mL versus 46.69 pg/mL and median 31.4 pg/mL versus 15.8 pg/mL, *p*-value 0.023) (Figure 2C).

### 3.4. CRP Versus IL-6 and Hemorrhage Versus Mortality

**Figure 3:**
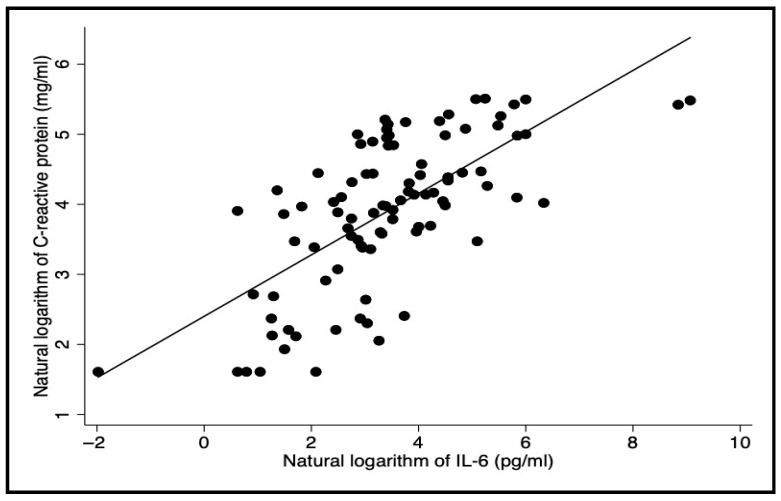
Correlation between sera Il-6 and C-reactive protein in patients with visceral leishmaniasis. Pearson correlation coefficient. r = 0.69; *p*-value < 0.001.

As expected, CRP levels increased with rising IL-6 levels, showing a strong correlation (r = 0.69; *p*-value < 0.001) (Figure 3). Out of 91 patients who tested for both markers, 84 (92.3%) had a positive result for at least one test. Neither CRP nor IL-6 were significantly correlated with Kala-Cal^®^ (r = 0.18 and 0.12, respectively. Hemorrhage occurred in nine patients, and of the eight who died, five had hemorrhage (*p*-value < 0.001).

### 3.5. CRP, IL-6, and Kala-Cal^®^ as Biomarkers of VL Mortality

For predicting death, CRP showed an area under the curve (AUC) of 0.85 (95% confidence interval (CI) 0.59; 1.00), while IL-6 showed an AUC of 0.87 (95% CI 0.73; 1.00). The AUC for the Kala-Cal^®^ prognostic score was 0.83 (95% CI 0.62; 1.00). The three AUCs were not statistically different. The AUC in patients with HIV could not be assessed due to the small number (three) of patients who died.

Table 2 summarizes the performance of CRP and IL-6 at two cut-offs. Youden’s J statistic for CRP was 145.9 mg/L, but adjusting the cut-off to 148 mg/mL did not change the receiver operating characteristics, yielding a sensitivity of 0.86 (95% CI 0.42; 1.00) (6/7 deceased) and specificity of 0.86 (95% CI 0.77; 0.93) with 74/86 CRP-negative surviving patients (*p*-value < 0.001). For easier recall, a cut-off of 150.0 mg/L, resulted in a sensitivity 0.71 (95% CI: 0.29; 0.96) (5/7 deceased), and specificity of 0.88 (95% CI 0.80; 0.94) with 76 negative results out of the 86 who survived (*p*-value = 0.001). Lowering the cut-off to 100 mg/L improved sensitivity to 0.86 (95% CI: 0.42–1.00) (6/7 deceased), but specificity dropped to 0.78 (95% CI: 0.68–0.86) with 67/86 survivors (*p*-value = 0.001). Youden’s J statistic for IL-6 was 89.7 pg/mL. Rounding the value to 90 pg/mL yielded a sensitivity of 0.88 (95% CI: 0.47–1.00) (7/8 deceased) and a specificity of 0.81 (95% CI: 0.71–0.88) (75/93 patients) (*p*-value < 0.001). When the cut-off was lowered to 50 pg/mL for easier recall, sensitivity remained at 0.88 (95% CI: 0.47–1.00) (7/8 deceased), but specificity decreased to 0.70 (95% CI: 0.60–0.80) (65/93 patients) (*p*-value = 0.002).

The impact of CRP and IL-6 on post-test probability at two distinct cut-offs, using the Kala-Cal^®^ prognostic model to estimate the chance of death following clinical evaluation is shown in Table 3.

Positive test results increased a low clinical pre-test probability (1–3%) to a concerning level exceeding 10%—the observed mean mortality rate for visceral leishmaniasis. Probabilities above this threshold may warrant consideration of additional treatment approaches or referral to intensive care. Conversely, negative test results allow clinicians to monitor patients with Kala-Cal^®^ estimates up to 15–20% more confidently, while pre-test probabilities below 10% provide greater reassurance for continued standard management.

**Figure 4:**
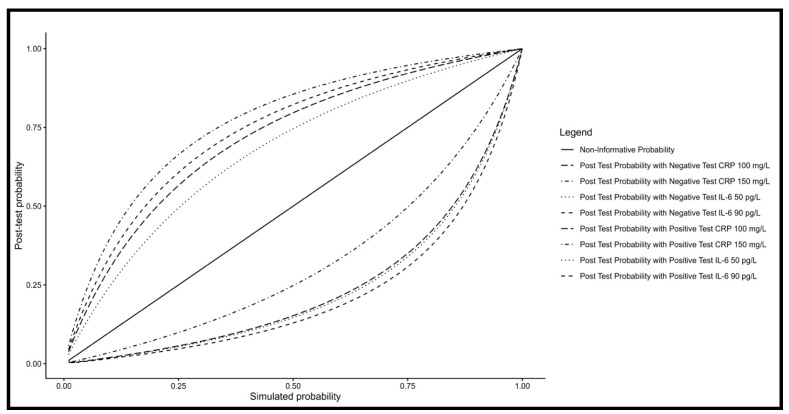
Simulation of post-tests probabilities according to progressive pre-test probabilities given by the Kala-Cal^®^ software.

Figure 4 presents a comparative analysis of post-test probabilities for CRP and IL-6 at selected cut-off values across a range of pre-test probabilities, based on simulation data. At lower pre-test probabilities, positive CRP (150 mg/L) and IL-6 (90 pg/L) results exerted the greatest impact, indicating a rapid increase in the probability of death. Although negative CRP results showed the poorest performance for ruling out the risk of death at the 150 mg/mL cut-off, negative test results showed to be useful for patient monitoring and follow-up.

## 4. Discussion

This in-hospital cohort confirms previous observations that CRP and IL-6 reach very high plasma concentrations in patients with visceral leishmaniasis prior to initiation of specific treatment. Our findings further demonstrate that elevated levels of these biomarkers are strongly associated with hemorrhagic manifestations, other clinical features of disease severity, and subsequent in-hospital mortality. These results support the concept that the pathophysiology of visceral leishmaniasis involves marked systemic inflammation, a pronounced acute-phase response, and activation of disseminated intravascular coagulation pathways. Both CRP and IL-6 showed good prognostic performance, particularly among patients classified as having low-to-intermediate risk at the initial clinical evaluation.

The sample was representative of New World VL, as seen by the higher proportion of men and children [4]. Also, mortality was similar to the mortality by the disease in Brazil [14]. However, patients with HIV were over-represented as compared to the national proportion of this coinfection [15]. On the other hand, as CRP and IL-6 did not vary according to HIV infection in this study population, it is unlikely that age has changed the representativeness of the study population.

The study indicates that the predictive value of CRP and IL-6 arises from their pathogenic roles and the correlation between them reflects a direct causal relationship, as IL-6 induces hepatic CRP production. After being synthesized by mononuclear cells upon stimulation by microbes’ pathogen-associated molecular patterns, IL-6 is released into the plasma, where it finds its receptor. One of its the main actions is to trigger the acute phase response by the liver via its membrane receptor on hepatocytes [13]. Transduction signs increase the synthesis of several of the so called acute-phase proteins. CRP is one of these, including some pro-coagulant proteins [16,17]. IL-6 also increases tissue factor expression in mononuclear and endothelial cells, triggering the extrinsic coagulation cascade, fueling disseminated intravascular coagulation, followed by consumption coagulopathy and hemorrhages, as seen in VL, bacterial sepsis and hemorrhagic fevers [18,19]. Indeed, bacterial infections account for most deaths in visceral leishmaniasis, likely by exacerbating the risk of fatal hemorrhagic complications. Thrombocytopenia in these patients results, at least in part, from platelet consumption, which further aggravates the splenic sequestration associated with hypersplenism [20,21]. Anemia is also mediated by the acute-phase response, particularly through hepcidin-induced inhibition of intestinal iron absorption and impaired iron recycling from macrophages [22,23].

The observed AUC values of 0.85 or higher indicate strong prognostic performance for mortality biomarkers. Notably, these values compare favorably with those reported for lactate and procalcitonin in predicting death among patients with bacterial sepsis [24,25]. Nevertheless, it is important to emphasize that no biomarker is decisive in isolation; their primary value lies in augmenting pre-existing clinical and epidemiological risk assessments. In visceral leishmaniasis, this baseline risk estimation is provided by the clinical status at the time of diagnostic suspicion during the initial hospital evaluation, with the corresponding pre-test probability generated by the Kala-Cal^®^ prognostic model.

In Brazil, the case-fatality rate of visceral leishmaniasis after diagnosis remains close to 10%. The main complications associated with death are hemorrhage and bacterial infections, with hemorrhagic events often precipitated by unrecognized infections. Although no specific therapy exists for disseminated intravascular coagulation, earlier identification of bacterial infections and timely initiation of antibiotic treatment may reduce case-fatality. Therefore, improved identification of patients at high risk of death has the potential to meaningfully reduce mortality.

In this study, risks of death up to 3–4% after clinical examination can rise to more than 10% if PCR and IL-6 are above the cut-offs, thus alerting the physician to look for early complications and define additional appropriate treatment, which may potentially reduce mortality. Clinical signs that lead to pre-test probability around 7–8% can rise to 20% to 30% if PCR or IL6 are positive, requiring immediate reevaluation, and decisions regarding of supporting treatments and referral of more appropriated medical support. Higher pre-test probabilities may not even require the tests since they already indicate immediate additional diagnosis and treatment decisions. Finally, negative results of patients with lower chance of death at the first clinical evaluation may result in more liberal medical attention, while those with up to 15% may just require a closer observation. The simulation helped us to see that the most informative option is IL-6 at the cut-off of 90 pg/mL, particularly for patients with the average mortality of around 10% seen in the country.

However, two additional points warrant consideration. First, only eight deaths were recorded, resulting in wide confidence intervals for sensitivity estimates and consequently limiting the robustness of the results, including the AUC. Replication of these findings in other settings and in studies with larger sample sizes is therefore necessary to confirm these preliminary observations. Second, our group recently published two studies in which IL-8, rather than IL-6, was significantly associated with mortality (AUC 0.75 vs. 0.60), and another study in which the AUC (0.67) was statistically significant but lower than that reported here [26,27]. The reasons for these discrepancies remain unclear, as the studies were conducted at different time periods despite being carried out at the same site. Random variation and time-dependent factors—such as disease duration at presentation, referral policies, place of residence, and other contextual variables—may have contributed to the observed differences. Currently, a larger dataset incorporating samples from multiple studies is being assembled to facilitate a pooled analysis aimed at more precisely defining the prognostic relevance of CRP, IL-6, and other inflammatory biomarkers in visceral leishmaniasis.

## 5. Conclusions

The findings support the targeting of systemic inflammation—a hallmark of VL pathogenesis—for the identification of mortality biomarkers. Both CRP and IL-6 emerged as promising biomarkers for fatal outcomes in VL. Their incorporation into initial clinical evaluations, whether in primary care or hospital settings, could enhance risk stratification and help address the growing case-fatality burden of VL in Brazil.

## Figures and Tables

**Table 1:** Characteristics of the 101 patients of the study population.

Variable	Number of Patients	Percent
Age groups (years)		
≤1	13	12.8
2–18	23	22.7
19–39	35	34.6
≥40	30	29.7
Sex		
Males	59	58.4
Females	42	41.5
HIV-1	35	34.6
Chance of death calculated with Kala-Cal^®^		
<20.0%	83	82.1
≥20.0%	18	17.9
In-hospital death	8	7.9

**Table 2:** Sensitivity and specificity of C-reactive protein and IL-6 for predicting death of patients with visceral leishmaniasis at different cut-offs.

Receiver Operational Characteristics	Cut-Offs			
	CRP * ≥ 150 mg	CRP ≥ 100 mg	IL-6 ≥ 90 pg	IL6 ≥ 50 pg
Sensitivity	0.71 (0.29; 0.96)	0.86 (0.42; 1.00)	0.86 (0.42; 1.00)	0.88 (0.47; 1.00)
Specificity	0.88 (0.80; 0.94)	0.78 (0.68; 0.86)	0.78 (0.68; 0.86)	0.70 (0.60; 0.80)

* C-reactive protein.

**Table 3:** Post-test probability of death associated with C-reactive protein and IL-6 results (at two cut-offs), as calculated by Kala-Cal^®^ software for varying pre-test probabilities.

		Post-Test Chance of Death (%)							
Pre-Test Chance of Death Estimated by Kala-Cal^®^ (%)	Number of Patients	CRP *				IL-6			
		Cut-Off 150 mg/mL		Cut-Off 100 mg/mL		Cut-Off 90 pg/mL		Cut-Off 50 np/mL	
		Positive Test (%)	Negative Test (%)	Positive Test (%)	Negative Test (%)	Positive Test (%)	Negative Test (%)	Positive Test (%)	Negative Test (%)
0.1	3	1	0	0	0	1	0	0	0
0.7	11	4	0	3	0	3	0	2	0
1.1	4	6	0	4	0	5	0	3	0
1.6	7	9	1	6	0	7	0	5	0
3.0	4	16	1	11	1	13	1	8	1
3.6	6	18	1	13	1	15	1	10	1
7.6	19	33	3	24	2	28	1	19	1
12.7	1	43	5	36	3	40	2	30	2
15.4	28	52	6	42	3	46	3	35	3
28.8	10	71	12	61	7	65	6	57	4
47.4	5	85	23	78	14	81	12	73	13
66.7	2	92	40	89	26	90	23	85	26
81.6	1	96	59	95	44	95	40	93	43

* C-reactive protein.

## Data Availability

The raw data supporting the conclusions of this article will be made available by the authors on request.
